# ADAMTS1 Supports Endothelial Plasticity of Glioblastoma Cells with Relevance for Glioma Progression

**DOI:** 10.3390/biom11010044

**Published:** 2020-12-31

**Authors:** Orlando Serrano-Garrido, Carlos Peris-Torres, Silvia Redondo-García, Helena G. Asenjo, María del Carmen Plaza-Calonge, José Luis Fernandez-Luna, Juan Carlos Rodríguez-Manzaneque

**Affiliations:** 1 GENYO, Centre for Genomics and Oncological Research: Pfizer/Universidad de Granada/Junta de Andalucía, Avenida de la Ilustración, 114, 18016 Granada, Spain; osbiotec@gmail.com (O.S.-G.); carlos.peris@genyo.es (C.P.-T.); silvia.redondo@genyo.es (S.R.-G.); helena.gomez@genyo.es (H.G.A.); mcarmen.plaza@genyo.es (M.d.C.P.-C.); 2 Faculty of Medicine, University of Panama, Ciudad Universitaria, Panamá 3366, Panama; 3 Molecular Genetics Unit, Hospital Universitario Marqués de Valdecilla, Avenida Valdecilla, s/*n*, 39008 Santander, Spain; joseluis.fernandezl@scsalud.es

**Keywords:** ADAMTS proteases, endothelial-like phenotype, extracellular metalloprotease, glioblastoma, plasticity

## Abstract

Gliomas in general and the more advanced glioblastomas (GBM) in particular are the most usual tumors of the central nervous system with poor prognosis. GBM patients develop resistance to distinct therapies, in part due to the existence of tumor cell subpopulations with stem-like properties that participate in trans-differentiation events. Within the complex tumor microenvironment, the involvement of extracellular proteases remains poorly understood. The extracellular protease ADAMTS1 has already been reported to contribute to the plasticity of cancer cells. Accordingly, this basic knowledge and the current availability of massive sequencing data from human gliomas, reinforced the development of this work. We first performed an in silico study of ADAMTS1 and endothelial markers in human gliomas, providing the basis to further assess these molecules in several primary glioblastoma-initiating cells and established GBM cells with the ability to acquire an endothelial-like phenotype. Using a co-culture approach of endothelial and GBM cells, we noticed a relevant function of ADAMTS1 in GBM cells leading the organization of endothelial-like networks and, even more significantly, we found a blockade of the formation of tumor-spheres and a deficient response to hypoxia in the absence of ADAMTS1. Our data support a chief role of this protease modulating the phenotypic plasticity of GBM.

## 1. Introduction

Gliomas are the most usual and lethal type of primary malignant tumors of the central nervous system. Indeed, the more advanced grade glioblastoma multiforme (GBM) (recently classified as IDH-wild type) has a very poor prognosis and a median overall survival of only 14–16 months in newly diagnosed patients [1,2]. GBM is characterized by its invasive properties and also they possess a very robust vascularization including abnormal blood vessels [3], favoring the frequent episodes of resistance to chemotherapy, radiotherapy and antiangiogenic drugs. It has been remarked the tremendous plasticity of tumor cells in GBM cases, supported by the existence of tumor cell subpopulations with stem cell-like properties [4]. There are relevant works supporting the trans-differentiation of these cancer stem cells to arise vascular endothelium and pericytes [5,6,7,8]. The acquisition of an endothelial-like (EL) phenotype was also introduced with the concept of vasculogenic mimicry [9], revealed in some tumors as an alternative mechanism of vascularization where tumor cells revert to a stem-like state and a consequent conversion to pseudo-endothelium [10].

The maintenance and behavior of all cells within the tumor is intimately linked to its tumor microenvironment (TME) where the extracellular matrix (ECM) plays a critical role [11]. In line with the high dynamism of the TME, extracellular proteases contribute to the alteration of multiple pathways during neoplasia progression. Among these proteases, studies on the first member of the ADAMTS (*A disintegrin and metalloprotease with thrombospondin motifs*) family already emphasized its contribution to achieve an EL phenotype [12]. More recently, the role of ADAMTS1 in modulating plasticity features has been reported in a melanoma model [13]. Common to the modulatory nature of these type of proteases, different reports presented a tumor suppressive activity for ADAMTS1 [14,15] and, on the contrary, there is evidence of its protumorigenic properties [13,16,17,18]. In fact, similar attributes have been stated for other ADAMTSs [19]. In the context of gliomas, ADAMTS4 and ADAMTS5 were found to be highly expressed in human GBMs [20], suggesting a contribution mediated by their activity on the proteoglycan brevican, highly present in the brain ECM [21]. Furthermore, we already suggested ADAMTS1 plays a role in glioma through its action on IGFBP2 (insulin-like growth factor-binding protein 2) [22] but without any functional outcomes involving the plasticity of GBM cells.

Nevertheless, the study of extracellular molecules affecting cancer cells plasticity is needed according to the limited understanding of these phenotypic alterations [23]. Although the exploration of the epithelial to mesenchymal transition has provided very relevant findings in the field of metastasis, its reverse mesenchymal to epithelial transition is less well known, and the acquisition of stem-like characteristics leading to de-differentiation and trans-differentiation processes remains still a big challenge to investigate.

Here, an initial in silico analysis of glioma datasets with a relevant number of patients allowed us to observe a positive correlation of ADAMTS1 and endothelial markers with glioma progression. Furthermore, the differentiation of several primary glioblastoma-initiating cells (GICs) and established GBM cells showed high levels of this protease and endothelial-related genes. While Matrigel-based assays confirmed the induction of an EL phenotype of GBM cells in the absence and presence of co-cultured endothelial cells, the inhibition of ADAMTS1 unveiled a previously unknown involvement of this protease supporting plasticity features of some GBM cells, including their blockade under hypoxia conditions.

## 2. Materials and Methods

### 2.1. Bioinformatic Analysis of Glioma Samples

Data from glioma samples were obtained from mRNAseq_693 dataset of the Chinese Glioma Genome Atlas (CGGA) and The Cancer Genome Atlas (TCGA) Glioblastomas (GBM) and Low Grade Gliomas (LGG) datasets. CGGA project provided RNA sequencing data of 693 glioma specimens from different grades (*n* = 188 for WHO II, *n* = 255 for WHO III and *n* = 249 for WHO IV; there is no grade data for 1 sample of this dataset). TCGA included RNA sequencing data of 652 glioma specimens from different grades (*n* = 243 for WHO II, *n* = 262 for WHO III and *n* = 145 for WHO IV; there are no grade data for 2 samples of this dataset). Various analyses were performed using the provided tools, such as gene expression, IDH status, correlation studies, and Kaplan-Meier survival curves. High and low expression cutoffs are determined by dividing the samples on the median.

### 2.2. Culture of Primary Tumor Neurospheres and GBM Cells and Generation of ADAMTS1-Knockout Cells

Glioblastoma-initiating cells (GICs) from tumors were achieved as described [24]. Briefly, tumor samples were enzymatically digested, and then tumor cells were resuspended in serum-free Dulbecco’s modified Eagle’s medium (DMEM)/F12 medium (Invitrogen, Carlsbad, CA, USA) containing human recombinant EGF (20 ng/mL; Sigma, St Louis, MO, USA), bFGF (20 ng/mL; Sigma-Aldrich, St. Louis, MO, USA), B-27 (20 μL/mL of medium; ThermoFisher Scientific, Waltham, MA, USA) and heparin (2 μg/mL), and plated at a density of 3 × 10^6^ live cells/60-mm plate. Primary neurospheres were detected within the first 2 weeks of culture and subsequently dissociated every 3–4 days to facilitate cell growth. Written consent from patients was obtained following requirements by the Research Ethics Board at the Valdecilla Hospital (Santander, Spain).

Human glioblastoma cell lines U87-MG, U251-MG, U373-MG and T98G were cultured in DMEM High Glucose with l-Glutamine (Biowest, Nuaillé, France), supplemented with 10% Fetal Bovine Serum (ThermoFisher Scientific, Waltham, MA, USA), and 1% of penicillin/streptomycin solution (Biowest, Nuaillé, France). Human umbilical vein endothelial cells (HUVECs) were plated on a 0.1% gelatin base and cultured with Endothelial Cell Growth Medium-2 (EGM^TM^-2) (Lonza, Basel, Switzerland). All cells were maintained under standard conditions (37 °C, 5% CO2 and 95% relative humidity). For hypoxia, cells were maintained during the entire experiment in a Whitley H135 Hepa Hypoxia Station at 1% oxygen.

The inhibition of ADAMTS1 in U87-MG and U251-MG cells was accomplished by a lentivirus-based CRISPR/Cas9 system as described [25]. After single cloning and expansion, cells were subjected to Sanger DNA sequencing (primer sequences in Supplementary Table S1) and Western Blot to confirm gene edition and inhibition, respectively. ADAMTS1 deficient cells are named U87-ATS1ko1, U87-ATS1ko2, U251-ATS1ko1, U251-ATS1ko2, and U251-ATS1ko3 throughout the manuscript.

### 2.3. Culture Assays

#### 2.3.1. Tumor-Sphere Assay

In total, 100,000 cells were seeded in a non-adherent plates with CSC medium (DMEM F12 supplemented with 1X B27™ (ThermoFisher Scientific, Waltham, MA, USA), 10 ng/mL Fibroblast Growth Factor-2 (Miltenyi Biotec, Bergisch Gladbach, Germany), and 10 ng/mL Epidermal Growth Factor (Miltenyi Biotec, Bergisch Gladbach, Germany). Medium was renewed every 4 days by low speed centrifugation (5 min, 800 rpm). Tumor-spheres were allowed to develop for 21 days (primary spheres). To generate secondary spheres, primary ones were disaggregated with trypsin and seeded again for 2 additional weeks. Images were captured with an Axio Vert microscope (A-Plan 5×/0.12 objective, Zeiss, Oberkochen, Germany), and evaluated with Carl Zeiss ZEN 2.3 SP1 (black) software. Quantification of spheres area was performed using ImageJ software, evaluating random quadruplicates.

#### 2.3.2. Differentiation of Neurospheres

To promote differentiation of GIGs and GBM cell lines, neurospheres were cultured with DMEM/F12 and DMEM, respectively, in adherent plates in the presence of 10% fetal calf serum for the indicated time intervals, according to [24].

#### 2.3.3. Matrigel-Based Assays

In total, 35 µL/well of Matrigel (Corning, NY, USA) were dispensed in a 96-well plate kept on ice to avoid gelling. After Matrigel gelling, 100 µL of serum-free medium were added to each well. Finally, 100 µL of serum-free medium containing cells were added. Follow-up was performed by taking pictures at various time points (Axio Vert microscope, A-Plan 5×/0.12 objective, Zeiss, Oberkochen, Germany). If appropriate, 24 h pictures were subjected to WimTube analysis (Wimasis, Córdoba, Spain) as indicated [26].

For co-cultures, cells were previously infected with lentivirus carrying EGFP (for GBM cells) and DsRed (for HUVECs) to approach their visualization (both lentiviral vectors were a gift from Dr F Martin, GENYO). Cells were seeded at a 1:1 ratio in Matrigel and evaluated by microscopy as above.

For invasion sprouting assays, 45 µL/well of Matrigel were used in a 96-well format, with 100 µl serum-free medium with 1% penicillin/streptomycin solution. A tumor-sphere was placed in each well and its progression was followed after 24 h according to Vinci et al. [27]. Images were captured as above, and the number and length of sprouts were quantified using the WimSprout analysis (Wimasis, Córdoba, Spain).

#### 2.3.4. Clonogenic Assay

Cells were trypsinized, washed and counted. Cells to be used were embedded in a 0.7% agarose solution containing DMEM 20% fetal bovine serum and 2% penicillin/streptomycin. Then, 1000 cells/well were seeded in 6-well plates and maintained at 37 °C. Then, 14 days later, supernatant was carefully removed, and cells were fixed and stained using crystal violet (20% ethanol and 0.5% crystal violet in water). Then, wells were washed with distilled water until discolored for their analysis and counting of colonies.

### 2.4. RNA Isolation and Quantitative RT-PCR

Total RNA was extracted from GICs and GBM cells using the NucleoSpin^®^ RNAII kit (Macherery-Nagel, Duren, Germany). cDNA was synthesized with iScript™ cDNA Synthesis Kit (Bio-Rad, Hercules, CA, USA). qPCR reactions were performed with Fast SYBR™ Green Master Mix (Applied Biosystems, Waltham, MA, USA), using a 7900HT Fast Real-Time PCR (Applied Biosystems, Waltham, MA, USA) machine. qPCR representations show the 2^(ΔΔCt)^. As housekeeping genes, *ACTB* was normally used excepting determinations in GICs that used *B2M*. All primer sequences are in Supplementary Table S1. Every run included triplicates per sample.

### 2.5. Western Blot Analysis

To evaluate secreted proteins, we analyzed conditioned medium (CM) from cells. For that, attached cells were washed repeatedly with phosphate buffered saline (PBS), and then maintained with medium without serum during 24 h. At this point CM was collected, centrifuged at 1200 rpm for 5 min to eliminate debris, and then concentrated with StrataClean resin (Agilent Technologies, Santa Clara, CA, USA). Protein extract was then resolved by SDS-PAGE and transferred to Polyvinylidene difluoride (PVDF) membranes (Bio-Rad, Hercules, CA, USA). Membranes were stained with a Red Ponceau solution to visualize loaded proteins. Then, they were blocked with 5% low-fat milk and incubated with a sheep anti-human ADAMTS1 antibody (AF5867, R&D Systems, Minneapolis, MN, USA). After incubation with the appropriate peroxidase-conjugated secondary antibody, signal was detected with the Amersham ECL Prime Western Blotting Detection Reagent (GE Healthcare Life Sciences, Marlborough, MA, USA) in an ImageQuant LAS4000 (GE Healthcare Life Sciences, Marlborough, MA, USA).

### 2.6. Statistical Analysis

Statistical analyses were performed using GraphPad Prism 8 (GraphPad software Inc., San Diego, CA, USA). For representations derived from CGGA and TCGA platforms, datasets were downloaded and statistical results were confirmed as consistent with those from CGGA and TCGA webtools. Pearson correlation analyses were directly obtained from CGGA. Except when indicated, graphs represent mean ± SEM (standard error of the mean), and unpaired *t* tests were performed to compare means of experimental groups.

## 3. Results

### 3.1. In Silico Analysis of ADAMTS1 Gene Expression in Human Gliomas Shows Its Importance and Correlation with Endothelial Features

**Figure 1:**
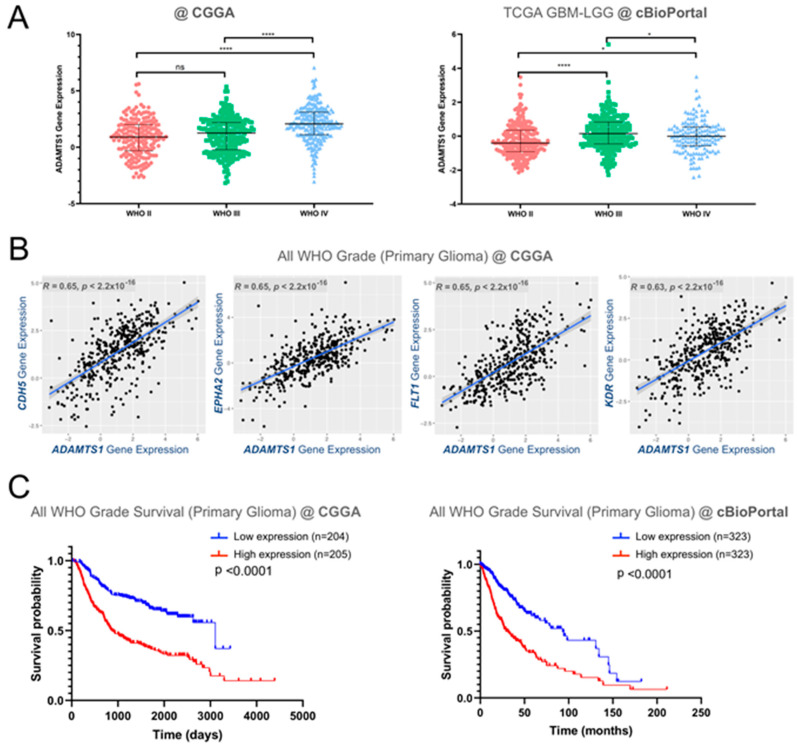
In silico analysis of *ADAMTS1* and endothelial markers in glioma samples from Chinese Glioma Genome Atlas (CGGA) and The Cancer Genome Atlas (TCGA) glioblastoma multiforme- Low Grade Gliomas (GBM-LGG) projects. (**A**) Graph representing *ADAMTS1* gene expression among different grades of glioma according to WHO classification, analyzing both CGGA and TCGA GBM-LGG datasets. TCGA data are analyzed with cBioPortal platform (****, *p* < 0.0001; *, *p* < 0.05 in unpaired *t*-test, using WHO II patients as control. Median and interquartile range are indicated for each group); (**B**) Scatter plots representing correlation analyses between gene expression levels of *ADAMTS1* and endothelial-related genes *CDH5, EPHA2, FLT1* and *KDR* (*R* = Pearson correlation coefficient); (**C**) Kaplan–Meier survival curves for low and high gene expression levels of *ADAMTS1* considering all glioma grades, in both CGGA and TCGA GBM-LGG datasets.

Although previous results already supported a putative role of ADAMTS1 in GBM [22], the new availability of data of a relevant number of patients, such as the CGGA and TCGA GBM-LGG projects, allowed us to approach a deeper and relevant in silico-based assessment. First of all, we observed a positive correlation between advanced glioma stages and an increasing *ADAMTS1* gene expression in both datasets including a total of 1342 gliomas (Figure 1A). Accordingly, the analysis of *ADAMTS1* expression separating mutant and wild type (WT) IDH status confirmed significant higher levels of this protease in WT IDH gliomas, as the vast majority of superior grade IV gliomas do not present these mutant forms [2] (Supplementary Figure S1A,B). In line with the relevance of angiogenesis mechanisms during glioma progression and previous findings that remarked the actions of ADAMTS1 in endothelial and vasculature-related events [12,18,28], we looked at the correlation of *ADAMTS1* with endothelial factors. Significantly, we found positive correlations between its expression and a panel of endothelial markers (*CD34, CDH5, ENG, EPHA2, FLT1* (VEGFR1), *KDR* (VEGFR2)) that have already been described as poor prognosis factors in GBM [29,30,31,32,33] (Figure 1B and Supplementary Figure S1C). Indeed, this positive correlation seemed in agreement with the involvement of trans-differentiated tumors cells in glioblastoma resistance to radiotherapy [34].

According to these observations we analyzed the relevance of ADAMTS1 for the survival of glioma patients. Significantly, Kaplan–Meier survival curves revealed a positive relationship between high levels of *ADAMTS1* and a worse prognosis of the disease including all grades and evaluating both CGGA and TCGA data (Figure 1C). The separate analyses by grades confirmed the worse progression of high *ADAMTS1* expressers for grades II and III, but not for advanced grade IV GBMs (Supplementary Figure S1D).

### 3.2. Expression of ADAMTS1 and Endothelial-Related Genes Is Significant in Primary and Differentiated GICs and GBM Cells

**Figure 2:**
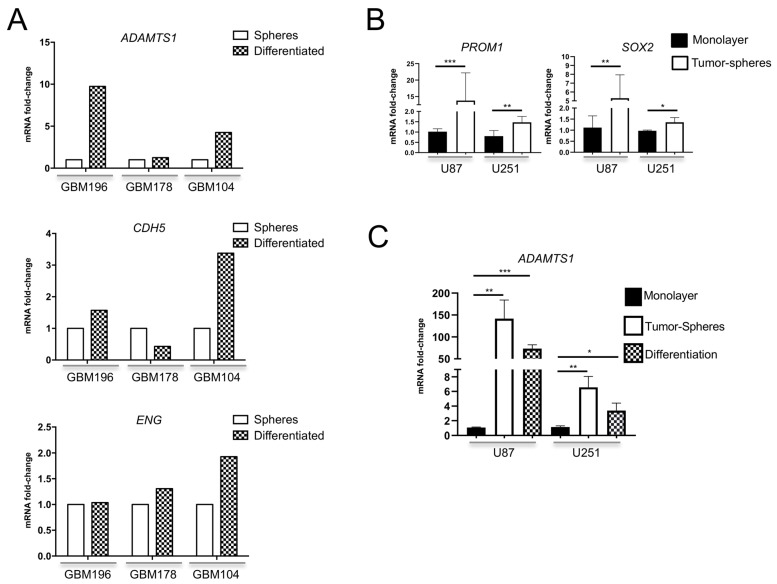
Evaluation of *ADAMTS1* gene expression and plasticity in GICs and GBM cell lines. (**A**) Graphs representing mRNA fold change expression of *ADAMTS1*, *CDH5* and *ENG* in independent GICs as spheres and undergoing differentiation (standard deviation is not included in these graphs as they represent individual tumor samples); (**B**) Graph representing mRNA fold change expression of stemness markers *PROM1 and SOX2* in monolayer cultures and tumor-spheres derived from U87-MG and U251-MG cells (*n* ≥ 3 samples for all cell lines); (**C**) Graphs representing mRNA fold change expression of *ADAMTS1* in monolayer cultures, tumor-spheres, and differentiation status, derived from U87-MG, and U251-MG cells (*n* ≥ 3 samples for all cell lines). **, *p* < 0.001; *, *p* < 0.01; and *, *p* < 0.05.

We assessed gene expression of *ADAMTS1* in several GIC cultures generated from fresh human GBM specimens (GBM196, GBM178 and GBM104). As described [24], these GICs form characteristic and renewable spheres being able to proliferate indefinitely in this format. For the purpose of this study, we also induced differentiation of GICs by the addition of fetal calf serum as reported [24], mimicking their in vivo evolution. *ADAMTS1* expression was detectable in all GICs tested and, importantly, it was induced in 2 out of 3 of these GICs at their differentiated status (Figure 2A, top graph). To evaluate their intrinsic endothelial-like properties, our analyses also revealed a relevant expression of endothelial-related genes such as *CDH5* and *ENG* in GICs (Figure 2A, medium and bottom graphs), with a tendency to be induced during differentiation in a similar pattern to *ADAMTS1*.

In agreement with previous reports that recognized tumor plasticity in GBM cell cultures, including the capacity to generate tumor spheres, we approached the well-established U87-MG and U251-MG cells. First, our evaluations revealed an increased gene expression of key molecules such as *PROM1 (CD133)* and *SOX2* in tumor spheres derived from these cells (Figure 2B) whose role in endothelial trans-differentiation has been reported [6,32]. Notably, we uncovered a significant induction of *ADAMTS1* protease in both U87-MG and U251-MG derived spheres in comparison with their respective monolayer cultures (Figure 2C). As for GICs, we also differentiated tumor spheres of GBM cell lines. Importantly, the expression of *ADAMTS1* remained high at this differentiated stage (Figure 2C). Finally, a comparison of expression values between GBM cell cultures and GICs disclosed very relevant levels of endothelial-related *CDH5* and *ENG* in the GICs material supporting the relevance of their intrinsic endothelial and plastic nature (Supplementary Figure S2).

### 3.3. ADAMTS1 Knockout Blocks the Formation of Tumor-Spheres and Sprouting

**Figure 3:**
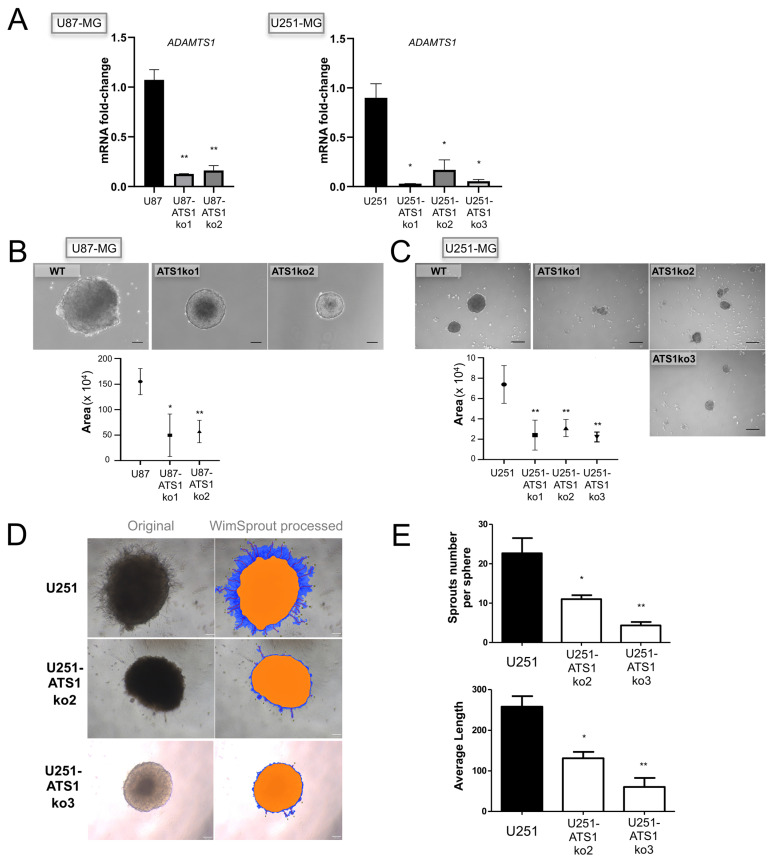
Effect of ADAMTS1 knockout in GBM cells. (**A**) Graph representing mRNA fold change expression of *ADAMTS1* in U87-MG, U251-MG, and their respective ATS1ko clones (*n* ≥ 3 for all cells) (**, *p* < 0.01; and *, *p* < 0.05. WT U87-MG and U251-MG cells were used as controls for statistical analyses, respectively); (**B**) Representative images of tumor-spheres derived from WT U87-MG and its ATS1ko clones, and graph representing their measures; (**C**) Representative images of tumor-spheres derived from WT U251-MG and its ATS1ko clones, and graph representing their measures; (**D**) Representative images (original and WimSprout processed) of 24 h sprouting tumor-spheres derived from WT U251-MG and U251-ATS1ko2 and ko3 clones; (**E**) graphs representing sprout number per sphere (top) and average length (bottom) according to WimSprout quantification of sprouting spheres as in (**E**) (*n* ≥ 3 samples for all cells) (**, *p* < 0.01; and *, *p* < 0.05).

In order to identify the relevance of ADAMTS1 in these processes, we inhibited its expression in U87-MG and U251-MG cells, according to the significant plastic behavior of these cell lines. In addition to confirm that *ADAMTS1* edition occurred properly, we checked gene expression levels of *ADAMTS1* in various inhibited clones (Figure 3A).

Then, making use of these ATS1ko cells, we approached their ability to form tumor-spheres following described methodology [35]. These assays revealed an evident blockade and difficulty of ADAMTS1-deficient cells to form spheres for both cell lines (Figure 3B,C), suggesting a chief role of the protease. In general, we observed smaller spheres, and particularly in U251-ATS1ko cells they were more irregular and with a high mortality; indeed, these tumor-spheres were unable to form secondary spheres, suggesting a noticeable loss of capacity for self-renewal.

In an attempt to measure their invasive capacity, we performed a sprouting and invasion assay analyzing primary tumor-spheres seeded in Matrigel as described elsewhere [27]. This assay provided an estimation of the invasive abilities of these tumor cells throughout the complex Matrigel matrix. In accordance with our current data, the sprouting ability of WT U251-MG cells at 24 h was clearly blocked in U251-ATS1ko cells (Figure 3D,E). This deficiency was confirmed by the unbiased quantification of number and length of sprouts (Figure 3E) using the WimTube webtool. Finally, the ability of U251-MG cells and its ATS1ko clones to generate in vitro colonies was determined using clonogenic assay as described [36], showing a decreased capacity in the absence of ADAMTS1 (Supplementary Figure S3). Likewise, these experiments support a pro-invasive and pro-tumorigenic activity for the protease ADAMTS1.

### 3.4. ADAMTS1 Plays a Role in Determining the Endothelial-Like Phenotype of GBM Cells

**Figure 4:**
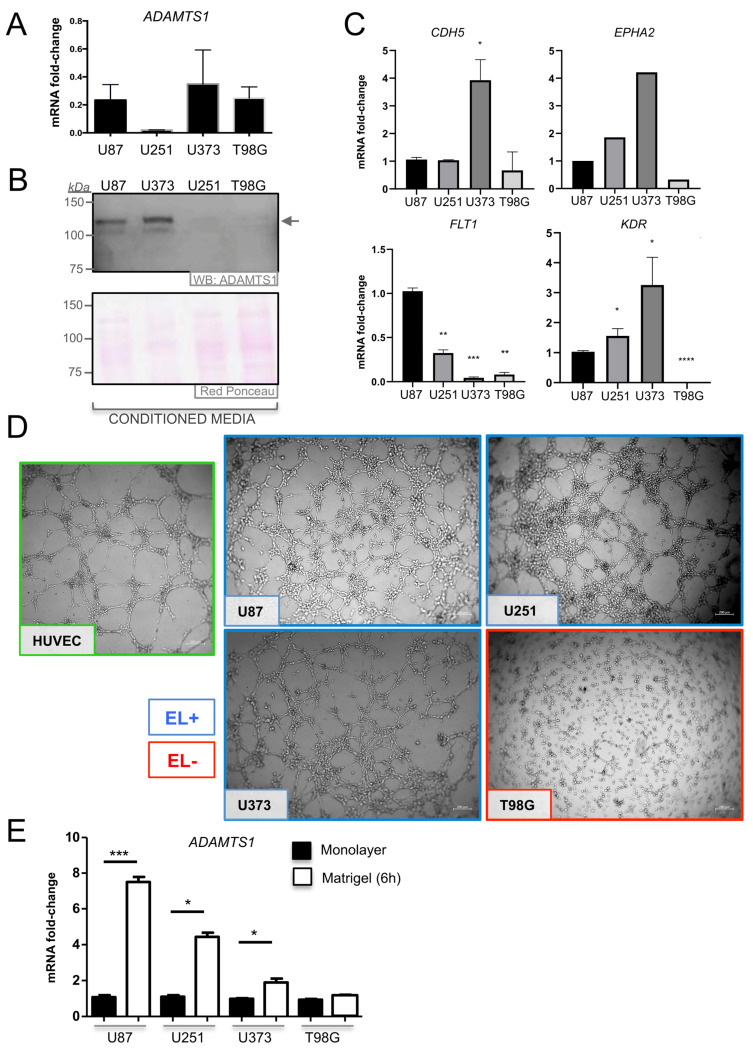
Analysis of ADAMTS1 in GBM cells and evaluation of their endothelial-like properties. (**A**) Graph representing mRNA fold change expression of *ADAMTS1* in GBM cells: U87-MG, U251-MG, U373-MG and T98G (values are relative to U87-MG cell line) (*n* ≥ 6 for all cell lines); (**B**) western blot analysis of ADAMTS1 in conditioned media of GBM cells. Black arrow points to full-length (FL) ADAMTS1. Red Ponceau staining was used as loading control; (**C**) graphs representing mRNA fold change expression of *CDH5*, *EPHA2*, *FLT1* and *KDR* in GBM cells (values are relative to U87-MG cell line) (*n* ≥ 3 for all genes except *EPHA2*, *n* = 1); (**D**) representative images of 3D Matrigel-based assay of HUVEC (green square) and GBM cells, 24 h after seeding (white scale bar = 200 µm); EL+ (blue square) and EL− (red square) cells are indicated; (**E**) graph representing mRNA fold change expression of *ADAMTS1* in GBM cells as monolayer (black bar) or in Matrigel assay (white bar) (*n* ≥ 4 for all conditions). (****, *p* < 0.0001; ***, *p* < 0.001; **, *p* < 0.01, and *, *p* < 0.05).

In addition to U87-MG and U251-MG, we evaluated gene and protein expression of ADAMTS1 in two more recognized human glioblastoma cell lines (U373-MG and T98G). With variations, all cell lines expressed ADAMTS1 (Figure 4A,B). According to our initial interest in the capacity of GBM cells to trans-differentiate to endothelial-like cells, we evaluated the expression of endothelial-related genes. Although some of the tested markers were undetectable by quantitative PCR, we detected genes such as *CDH5, EPHA2, FLT1* and *KDR* (Figure 4C), remarking the significant lowest expression of all these genes in T98G cells (Figure 4C). Then, we performed the established Matrigel assay to characterize their endothelial-like phenotype [37,38], including HUVECs as positive controls. Noticeably, we observed the positive behavior of U87-MG, U251-MG and U373-MG cell lines (cataloged as EL+), and the absence of an EL phenotype by T98G (cataloged as EL−) (Figure 4D). This distinct performance of T98G cells correlated with its lower expression of endothelial genes in comparison with EL+ cells (Figure 4C). We evaluated how *ADAMTS1* gene expression is affected during the process of induction of an EL phenotype in Matrigel. Very importantly, compared with cells under standard monolayer culture, we observed a clear and significant induction of *ADAMTS1* expression in all cell lines with an EL+ phenotype (U87-MG, U251-MG and U373-MG) but no alteration in the EL− T98G cells (Figure 4E).

**Figure 5:**
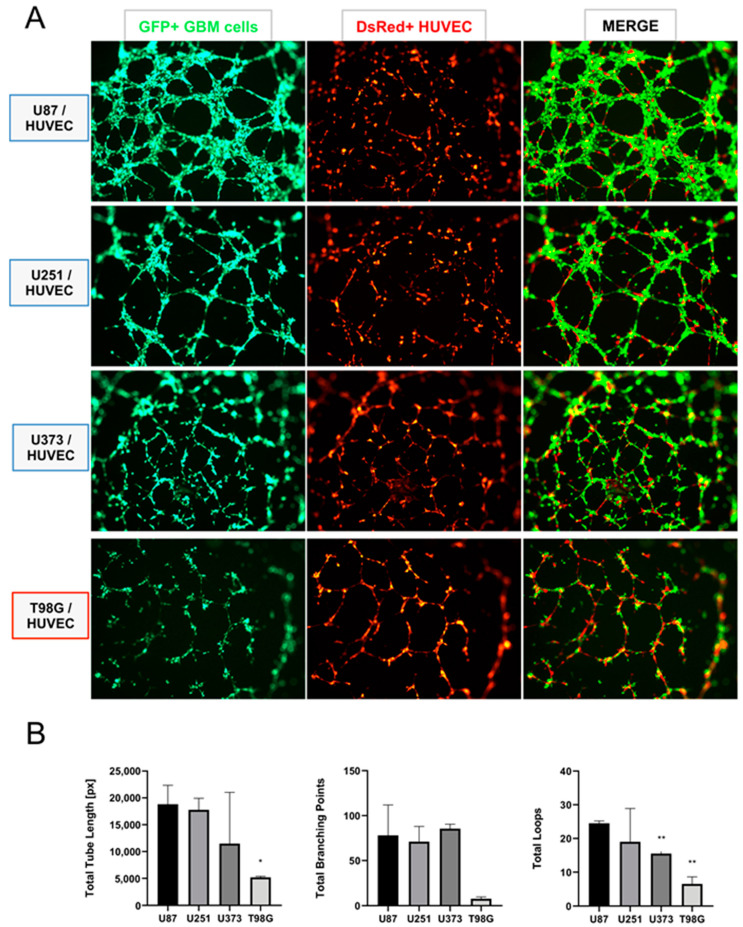
Behavior of GBM cells and HUVECs under co-culture conditions. (**A**) Representative images of a co-culture Matrigel assay containing GBM cells: U87-MG, U251-MG, U373-MG and T98G, from top to bottom (first column, green), co-cultured with HUVECs (second column, red). Third column is the resulting merge of GBM and HUVECs (associated images at time 0 are included in supplementary Figure S4); (**B**) graphs representing the WimTube quantification of several parameters comparing GBM cells under co-culture conditions: total tube length, total branching points and total loops (*n* ≥ 3 images for all cell lines) (**, *p* < 0.01; and *, *p* < 0.05. U87-MG cultures were used as control for statistical analyses).

These results support the contribution of the protease ADAMTS1 during the acquisition of an EL phenotype in the Matrigel assay. Therefore, to further study the significance of EL features of GBM cells, we performed their co-culture in Matrigel with genuine endothelial cells, specifically with HUVECs here, widely recognized by their ability to form a characteristic network (Figure 5 and Supplementary Figure S4). To distinguish between populations, GBM cells were labeled with GFP and HUVECs with *DsRed* (see Materials and Methods). Very interestingly, using a 1:1 ratio, we observed that the co-culture of HUVECs with U87-MG, U251-MG and U373-MG (classified as EL+) (Figure 5A, first to third rows), showed a dominance of GBM cells (first column, green channel) leading the endothelial-like networking, while HUVECs acted supporting such structure but as a secondary player (second column, red channel). In contrast, the co-culture of HUVECs with T98G cells (classified as EL−) (Figure 5A, fourth row) revealed a main structure leaded by HUVECs (second column, red channel). This observation is also visualized at the merge column, where we observed the prevailing green EL+ GBM cells at the three top rows, versus the dominant red HUVECs at the last row, co-cultured in this case with EL− T98G cells. To gain a quantitative perspective, we evaluated the consistency of EL+ structures of GBM cells under these co-culture conditions. We used the non-biased WimTube tool to analyze EL features (Figure 5B). Indeed, this inquiry revealed a significant decreased efficiency of T98G cells in comparison with the rest of GBM cell lines. Likewise, these assays confirmed the ability of some GBM cells to behave in an EL fashion including an intimate interaction with primary endothelial cells.

### 3.5. ADAMTS1 Knockout Partially Affects In Vitro Endothelial-Like Properties

**Figure 6:**
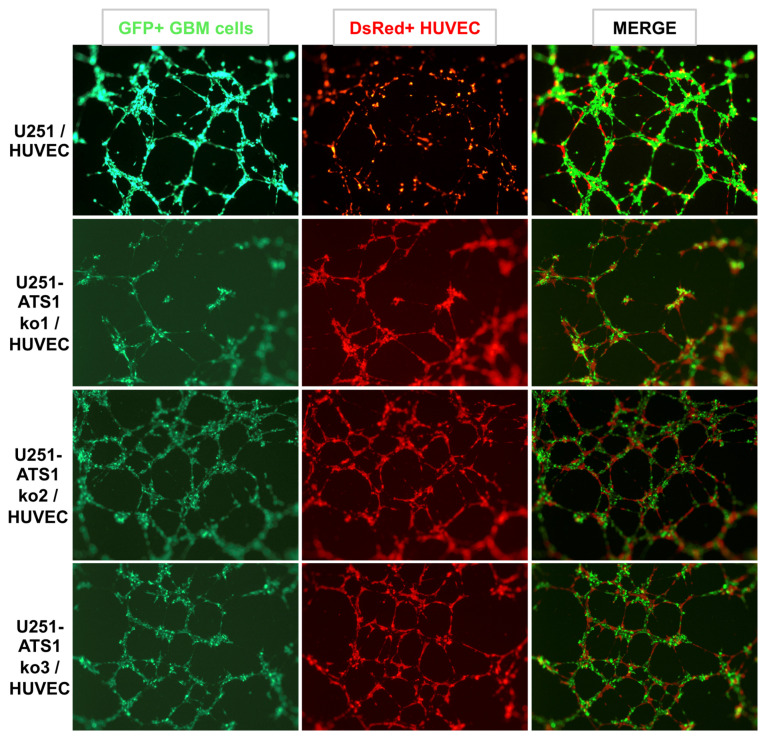
Effect of ADAMTS1 knockout in GBM cells co-cultured with HUVECs. Representative images of a co-culture Matrigel assay containing WT U251-MG (top row) or 3 U251-ATS1ko clones (second to fourth rows), co-cultured with HUVECs (second column, red). Third column is the resulting merge of U251 and HUVECs.

A first evaluation of the endothelial-like phenotypic properties of U87- and U251-ATS1ko cells in Matrigel did not indicate relevant differences in comparison with WT equivalents (data not shown). However, the performance of co-culture experiments with HUVEC, as showed above (Figure 5), did show interesting results for U251-ATS1ko cells. Comparing WT U251-MG and its U251-ATS1ko counterparts, first we confirmed that WT U251-MG leaded the endothelial-like network (Figure 6, first row). However, in the absence of ADAMTS1 in U251-ATS1ko cells, HUVECs gained a chief and prominent contribution to the endothelial networking (Figure 6, second to fourth rows). To remark that ATS1ko cells still maintain a relevant presence in the main structures, although showing some deficiencies.

### 3.6. ADAMTS1 and ENG Are Induced by Hypoxia in GBM Cells and Its Inhibition Blocks PROM1 Stemness Marker

**Figure 7:**
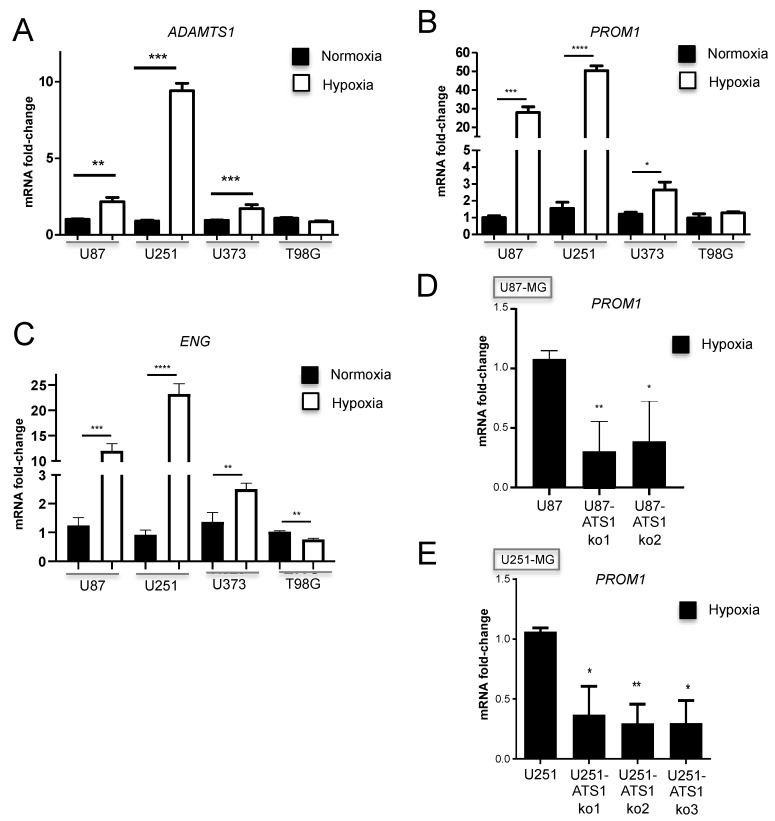
Evaluation of GBM cells under hypoxia and effect of ADAMTS1 knockout. (**A**–**C**) Graphs representing mRNA fold change expression of *ADAMTS1* (**A**), *PROM1* (**B**), and *ENG* (**C**) in normoxic and hypoxic cultures of GBM cells: U87-MG, U251-MG, U373-MG and T98G; (**D**) graph representing mRNA fold change expression of *PROM1* in hypoxic cultures of WT U87-MG and U87-ATS1ko clones; (**E**) graph representing mRNA fold change expression of *PROM1* in hypoxic cultures of WT U251-MG and U251-ATS1ko clones. (*n* ≥ 3 samples for all cell lines and conditions) (****, *p* < 0.0001; ***, *p* < 0.001; **, *p* < 0.01; and *, *p* < 0.05).

It has been described that critical hypoxia conditions induce processes of trans-differentiation in GBM [7]. Consistent with our work, now we included studies using hypoxia to see if it regulates ADAMTS1 and its relationship with stem-like features. Significantly, our analysis verified that *ADAMTS1* is induced in hypoxic conditions in 3 of the 4 tested GBM cell lines (Figure 7A); indeed, such activation occurred in U87-MG, U251-MG, and U373-MG, but not in T98G, in full agreement with our results showed here, where the classification of EL+ and EL− cells gathers with the induction of *ADAMTS1* under different conditions. We also evaluated the expression of the *PROM1* stemness marker in these cell lines, confirming its induction by hypoxia in EL+ cells U87-MG, U251-MG and U373-MG, but not in T98G (Figure 7B). Finally, with the rationale of investigating the presence of endothelial markers in GBM cells and their regulation by hypoxia conditions, we found a significant induction of endothelial-related *ENG*, recently suggested as a relevant biomarker in glioblastoma [33] and, in fact, nicely found in our GICs (Figure 2A and Supplementary Figure S2). In line with our finding with *ADAMTS1* and *PROM1*, *ENG* appears upregulated in hypoxic U87-MG, U251-MG and U373-MG but not in T98G cells (Figure 7C), even there is a down-regulation in this last cell type.

Finally, to verify that ADAMTS1 has a predominant role in the trans-differentiation and plasticity of GBM cells, we tested the ability of U87- and U251-ATS1ko clones to be activated under hypoxia. With this purpose we evaluated *PROM1* expression under such conditions, revealing its significant lack of induction in comparison with WT U87-MG and U251-MG cells (Figure 7D,E, respectively). This final result suggests a putative role of ADAMTS1 inducing stem-like features in GBM.

## 4. Discussion

Recent advances are allowing a better comprehension of the multiple players within the convoluted tumor microenvironment of gliomas in general and advanced GBMs in particular. However, the phenotypic characteristics of these tumors still remark on the necessity to investigate their high plasticity, sustained by the existence of cancer stem-like cells with the ability to acquire endothelial-like features [4,5,6,7,8]. Now, we focus our study in the specific contribution of the extracellular protease ADAMTS1 in glioma, first using in silico analyses of public available RNA-Seq datasets, and second performing a series of experimental approaches with glioblastoma cells.

Significantly, our initial studies on two independent cohorts of 693 and 652 gliomas (accessible through the CGGA and TCGA projects, respectively) showed an increased gene expression of *ADAMTS1* in more advanced glioma grades, and the overall survival data also supported the impact of this protease as a bad prognosis factor. Furthermore we showed the positive correlation of *ADAMTS1* with endothelial markers, such as *CD34*, *CDH5*, *ENG*, *EPHA2*, *FLT1* and *KDR*, already reported as bad prognosis in GBM [29,30,31,32,33]. However, a parallel analysis separating the different grades confirmed the association of *ADAMTS1* with worse prognosis just for grades II and III, but not for advanced GBM, probably due to the existence of additional and intricate parameters in this lethal group. Instead, the positive correlation at earlier stages of the disease is in line with additional outcomes of this work, as we will discuss below, mainly suggesting the role of ADAMTS1 as a regulator of the plasticity of tumor cells. An analogous scenario has been recently reported for uveal melanoma [13].

As we are interested in the acquisition of endothelial-like properties by GBM cells, we found the positive correlation of *ADAMTS1* with endothelial markers very encouraging, remarking that these genes are not discovered just in genuine endothelium, but they are also expressed in tumor cells. For example, *CDH5* signal has been found in GBM stem cell niches and it is induced by hypoxia [29], and further conventional markers of endothelium as *KDR*, *FLT1*, *PECAM*-1 and *CD34* have also been reported to contribute to the trans-differentiation of GBM cells [30,31,32]. Importantly, we revealed here the induction of *ENG* by hypoxia in EL+ GBM cells, highlighting the suggestive contribution of this molecule in glioma-related angiogenesis [33]. Therefore, ENG has been proposed as a biomarker in glioblastoma.

According to tumor plasticity already recognized for GBM cells [3,4,5,6,7], we included the analysis of primary neurospheres derived from GICs and their later differentiation. Several studies attribute an increased aggressiveness to these stem-like cells, so they could be responsible for the resistance to chemotherapy and radiotherapy [39,40]. Significantly, the expression of *ADAMTS1* was upregulated during differentiation of GICs with a similar pattern to endothelial-related *CDH5* and *ENG* genes, supporting the existence of an intrinsic endothelial-like nature within these tumor-promoting cells. Furthermore, our assays with GBM cell cultures revealed their plastic nature in partial agreement with primary GICs. Dealing with the deadly aggressive abilities of these tumors, we added the sprouting assay that allows for visualizing and quantifying the invasive properties of GBM cells [41]. These assays first revealed a significant induction of *ADAMTS1* in tumor-spheres in comparison with monolayer cultures, suggesting its relevance during this process. As we expected, the execution of these experiments with U87- and U251-ATS1ko cells confirmed its chief contribution as the evolution of their tumor-spheres was significantly blocked and, in fact, at least for U251-MG they were not able to get to secondary spheres as routinely occurs with WT cells. In addition, the sprouting and invasive capacities of these deficient cells were strongly compromised. Looking back to the data of ADAMTS1 as bad prognosis factor at early stages of glioma evolution, we postulate the major contribution of this protease in stem-like cells during such period, also according to the specific characteristics of the TME in that earlier tumor phase. Indeed, although *ADAMTS1* expression appears increased with tumor progression, the dynamism of the TME would involve new constituents that still require to be deeply investigated. In fact, to unveil the implication of ADAMTS substrates and to understand the impact in the TME of the exposition to therapies are major challenges [11].

Considering our work with GBM cell lines, although we found relevant levels of the protease ADAMTS1, the evaluation of EL properties in the Matrigel-based assay revealed the positive performance just of three of them (U87-MG, U251-MG and U373-MG, then catalogued as EL+). In parallel we were able to detect the induction of *ADAMTS1* gene expression in EL+ cells when they were in the process of forming the endothelial-like network, while no changes were observed in EL− T98G cells. Very interestingly, our additional studies under hypoxia conditions also presented the coincidence that *ADAMTS1* expression was induced in U87-MG, U251-MG and U373-MG; on the contrary, T98G cells were unresponsive once more. It appears clear that T98G, although originating from GBM, behave quite differently as their capacity to generate tumor-spheres was null in our hands, and neither *PROM1* nor *ENG* expression were induced under hypoxia conditions, as occurred with the rest of GBM cells. Surely, additional transcriptomic studies will help to comprehend these phenotypic differences and to determine the cooperation of multiple players for tumor plasticity.

In an attempt to mimic the interaction that tumor and endothelial cells undergo during tumor progression and trans-differentiation events, we approached the co-culture of endothelial and GBM cells. We already reported a bona fide interaction between endothelial and tumor cells in a similar setting [12]. Importantly, now we observed clear differences between the behavior of EL− and EL+ GBM cells. HUVECs showed the expected leading role to arrange the networking structure when co-cultured with EL− T98G cells. However, to our surprise, EL+ GBM cells displayed a remarkable dominance in this co-culture approach, possibly related with the strength of these cells in culture versus the more fragile attributes of primary endothelial cells. More significantly, the inhibition of ADAMTS1 in U251-MG cells altered the original capacities of this cell line in co-culture with HUVECs. These experiments showed that U251-ATS1ko cells failed, at least partially, to lead the formation of endothelial-like networks observed for WT cells, supporting the relevant role of ADAMTS1 to maintain an EL+ phenotype, now in close contact with endothelial cells.

## 5. Conclusions

In this report we disclose an increased expression of *ADAMTS1* according to grading of gliomas and it emerges as a bad prognosis marker for this neoplasia, mainly at its earlier stages. Our in silico studies showed a positive correlation of *ADAMTS1* with endothelial markers in glioma, association that we further corroborated performing in vitro co-culture assays of GBM and endothelial cells. A high expression of *ADAMTS1* and endothelial-related genes was demonstrated during the differentiation of primary GICs. Moreover, the genetic blockade of this protease in U87-MG and U251-MG cells confirmed its contribution to attain EL properties and revealed its requirement for tumor-spheres formation, glioma cell invasion and the response to hypoxia.

## Data Availability

Data is contained within the article or supplementary material.

## Supplementary Materials

The following are available online at https://www.mdpi.com/2218-273X/11/1/44/s1. Table S1: Sequences of used primers for genes of interest. Figure S1: Additional in silico analysis of *ADAMTS1* and endothelial markers in glioma samples from CGGA and TCGA GBM-LGG projects. Figure S2: Global comparison of gene expression values between GBM cell lines and GICs. Figure S3: Evaluation of clonogenic activity of U251-MG cells and its *ADAMTS1*-inhibited clones. Figure S4: Associates images at time 0 of co-culture assay presented in Figure 5A.
