# Supplementary material for: ADAMTS1 Supports Endothelial Plasticity of Glioblastoma Cells with Relevance for Glioma Progression
Source: Biomolecules. 2020 Dec 31;11(1):44. doi: 10.3390/biom11010044 (PMC7823850; doi:10.3390/biom11010044)
Supplement: Supplementary file 1 [file biomolecules-11-00044-s001.pdf]

Supplementary Table S1. Sequences of used primers for genes of interest.

Genes of interest and their use (qPCR or Sanger sequencing). Both forward and reverse sequences are indicated.

Use	Gene name	HGNC ID	Forward sequence (5'-3')	Reverse sequence (5'-3')
Sanger	*ADAMTS1*	HGNC:217	CTGCTACGAGCGGTGTCTC	GTGCCGGAGTAGAAGCAGTG
			CCCACAGGAACTGGAAGCATA	CCACTGCCGTGGAATTCTG
	ACTB	HGNC:132	GATGGCCACGGCTGCTT	AGGACTCCATGCCCAGGAA
	B2M	HGNC:914	GGACTGGTCTTTCTATCTCTTGT	TCTCGATCCCACTTAACTATCTTG
	CDH5	HGNC:1764	TGGTCACTCTGCAAGACATCAA	TCCTCAACAAACAGAGAGCCCACA

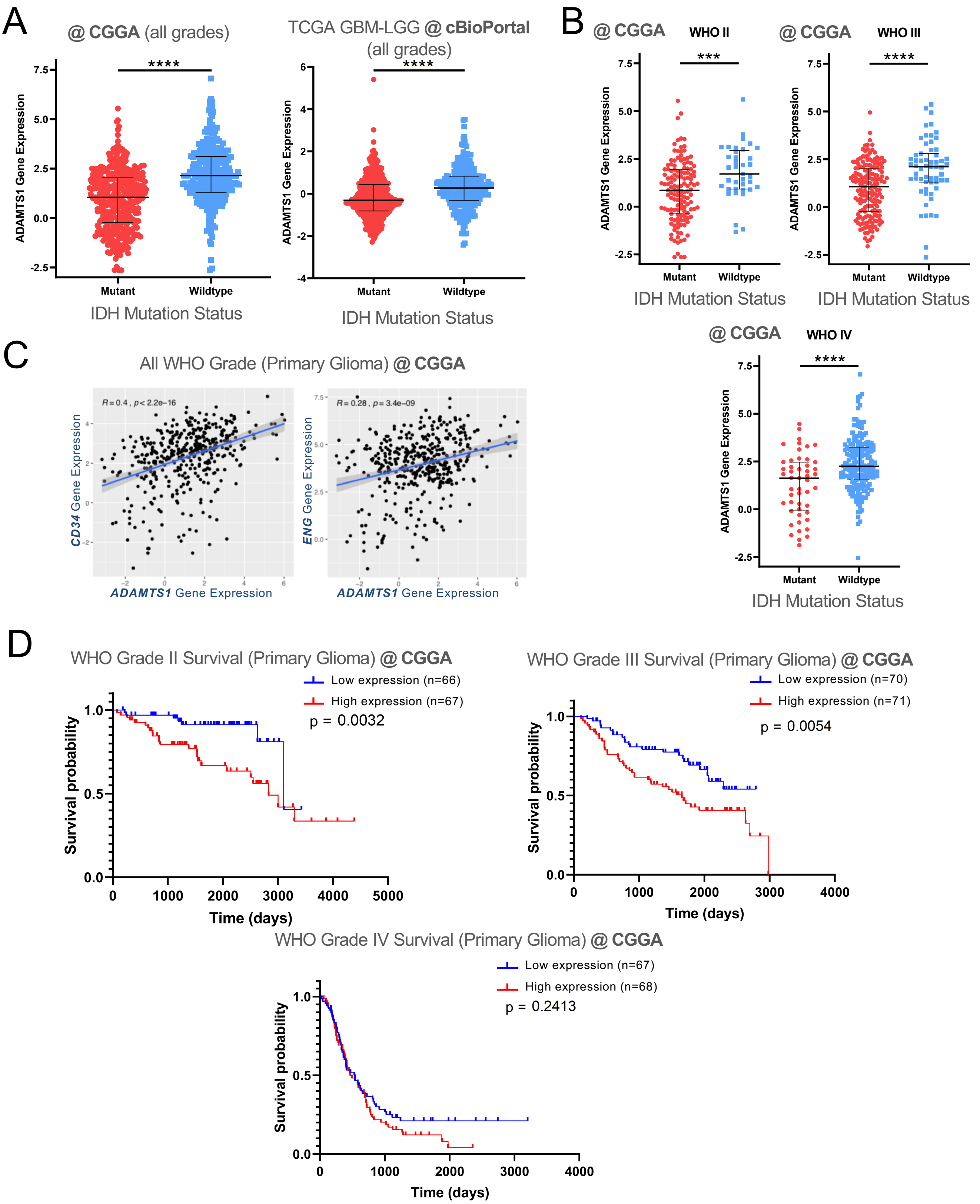

Supplementary Figure S1. Additional *in silico* analysis of *ADAMTS1* and endothelial markers in glioma samples from CGGA and TCGA GBM-LGG projects. **(A)** Graphs representing *ADAMTS1* gene expression in glioma samples (all grades) according to IDH mutation status, analyzing both CGGA (left) and TCGA GBM-LGG (right) datasets. TCGA data are analyzed with cBioPortal platform (****, p < 0.0001 in unpaired t test); **(B)** Graphs representing *ADAMTS1* gene expression among WHO grades II, III and IV in glioma samples of the CGGA project, according to IDH mutation status (****, p < 0.0001; ***, p < 0.001 in unpaired t test); **(C)** Scatter plots representing correlation analyses between gene expression levels of *ADAMTS1* and endothelial-related genes *CD34* and *ENG* (R=Pearson correlation coefficient); **(D)** Kaplan Meier survival curves for low and high gene expression levels of *ADAMTS1*, considering WHO grades II, III and IV in glioma grades of the CGGA project.

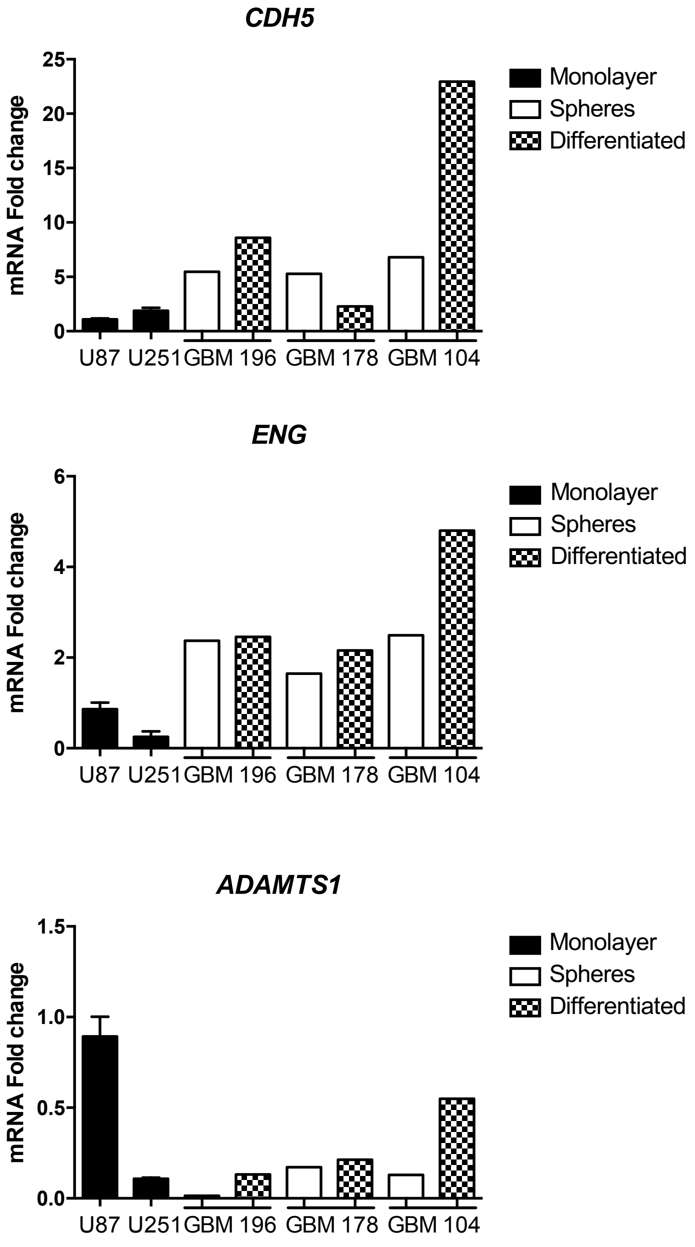

Supplementary Figure S2. Global comparison of gene expression values between GBM cell lines and GICs. Graphs represent mRNA fold change expression of *CDH5*, *ENG* and *ADAMTS1* genes in U87-MG and U251-MG cell lines, and GICs in their sphere and differentiated status. All values are relative to U87-MG cell line.

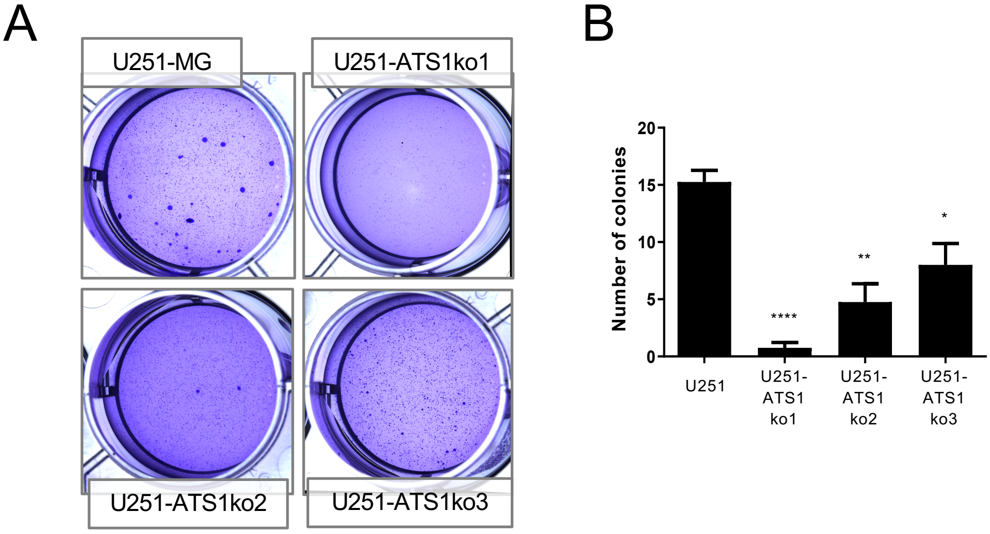

Supplementary Figure S3. Evaluation of clonogenic activity of U251-MG cells and its *ADAMTS1*–inhibited clones. **(A)** Representative microscopic images of clonogenic assay with U-251 MG cells, WT and ATS1ko clones ; **(B)** Graphs representing quantitative results of clonogenic assay expressed as total number of colonies/well (****, $p < 0,0001$; **, $p < 0,01$; and *, $p < 0,05$).

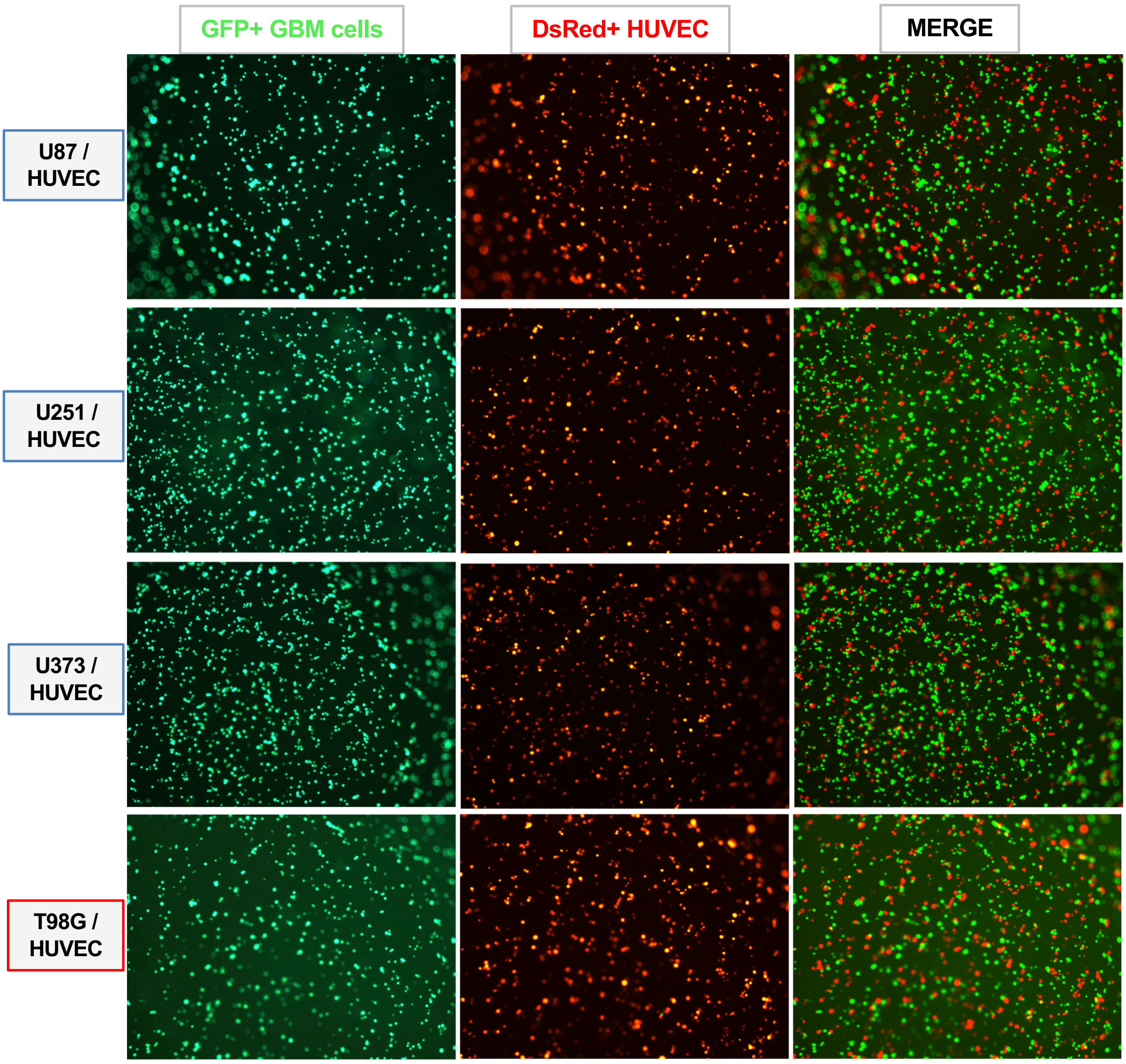

Supplementary Figure S4. Associated images at time 0 of co-culture assay presented in Figure 5A. They are representative images of co-culture Matrigel assay containing GBM cells: U87-MG, U251-MG, U373-MG and T98G, from top to bottom (first column, green), co-cultured with HUVECs (second column, red). Third column is the resulting merge of GBM and HUVECs.
